# Genome-wide scans using archived neonatal dried blood spot samples

**DOI:** 10.1186/1471-2164-10-297

**Published:** 2009-07-04

**Authors:** Mads V Hollegaard, Jonas Grauholm, Anders Børglum, Mette Nyegaard, Bent Nørgaard-Pedersen, Torben Ørntoft, Preben B Mortensen, Carsten Wiuf, Ole Mors, Michael Didriksen, Poul Thorsen, David M Hougaard

**Affiliations:** 1 Section of Neonatal Screening and Hormones, Statens Serum Institut, Copenhagen, DK-2300, Denmark; 2 Department of Epidemiology, University of Aarhus, Aarhus, DK-8000, Denmark; 3 AROS Applied Biotechnology A/S, Aarhus, DK-8000, Denmark; 4 Institute of Human Genetics, University of Aarhus, Aarhus, DK-8000, Denmark; 5 Department of Clinical Biochemistry, Skejby Sygehus, Aarhus, DK-8000, Denmark; 6 The National Centre for Register Based Research, University of Aarhus, Aarhus, DK-8000, Denmark; 7 Bioinformatics Research Center, University of Aarhus, Aarhus, DK-8000, Denmark; 8 Centre for Psychiatric Research, Aarhus University Hospital Risskov, Aarhus, DK-8000, Denmark; 9 Lundbeck A/S, Taastrup, DK-2630, Denmark; 10 Department of Epidemiology, Rollins School of Public Health, Emory University, Atlanta, GA, USA

## Abstract

**Background:**

Identification of disease susceptible genes requires access to DNA from numerous well-characterised subjects. Archived residual dried blood spot samples from national newborn screening programs may provide DNA from entire populations and medical registries the corresponding clinical information. The amount of DNA available in these samples is however rarely sufficient for reliable genome-wide scans, and whole-genome amplification may thus be necessary. This study assess the quality of DNA obtained from different amplification protocols by evaluating fidelity and robustness of the genotyping of 610,000 single nucleotide polymorphisms, using the Illumina Infinium HD Human610-Quad BeadChip. Whole-genome amplified DNA from 24 neonatal dried blood spot samples stored between 15 to 25 years was tested, and high-quality genomic DNA from 8 of the same individuals was used as reference.

**Results:**

Using 3.2 mm disks from dried blood spot samples the optimal DNA-extraction and amplification protocol resulted in call-rates between 99.15% – 99.73% (mean 99.56%, N = 16), and conflicts with reference DNA in only three per 10,000 genotype calls.

**Conclusion:**

Whole-genome amplified DNA from archived neonatal dried blood spot samples can be used for reliable genome-wide scans and is a cost-efficient alternative to collecting new samples.

## Background

Studies of genetic influence in complex disorders usually require extensive genome explorations of large cohorts. A major bottleneck, however, is access to DNA from well-characterised patients and healthy controls. This may be circumvented by use of archived residual blood samples from newborn screening programs, which in several countries engage the entire population. The blood is usually collected by heel-prick and applied on special filter paper, a proven robust and convenient medium for transport and storage [1]. Storage policies on residual neonatal dried blood spot samples (DBSS) vary internationally, but several countries store residuals in repositories for later research purposes [2-8]. Stored DBSS combined with relevant clinical information from medical registries thus constitute an ideal resource for large studies. This set-up enjoys the advantage of representing the entire population under a certain age and of avoiding practically any kind of selection. In addition substantial costs may be saved.

The Danish Neonatal Screening Biobank (DNSB) contains nearly 2 million DBSS from virtually every Dane born after 1982. It has recently been updated to meet the new general guidelines for the establishment and operation of biobanks [9]. Access to samples for research requires approval from the Scientific Ethical Committee System, the Data Protection Agency and the DNSB Steering Committee. In Denmark, all citizens have a unique person-identifying number that is used across all public registration systems, including the DNSB. Denmark also operates a well-established public health care system offering treatment to all citizens. Together this makes it possible to study the "entire country as a cohort" and makes the DNSB an ideal resource for studying common and complex genetic diseases in Caucasians [10]. The major challenge using the DBSS for such studies is however the small amount of blood available. In theory, the amount of genomic DNA (gDNA) that can be extracted from a 3.2 mm punch of a DBSS is about 60 ng [11]. In general, only one or two 3.2 mm punches per DBSS can be reserved for each project, which is scarcely enough to genotype multiple single nucleotide polymorphisms (SNP). This problem may be overcome by whole-genome amplification (WGA) of the gDNA. Previous studies have used whole-genome amplified DNA (wgaDNA) for genotyping, and with fair success, but in most cases the number of polymorphisms that can be tested has been limited [11-18].

In this study we investigate if a proper combination of DNA-extraction and WGA procedure can produce wgaDNA samples suitable for 610,000 SNP genome-wide scan using the Illumina Infinium HD Human610-Quad BeadChip. Neonatal DBSS stored for 15 to 25 years in the DNSB are employed, and as reference is used high-quality gDNA samples recently obtained from the same individuals. Two different WGA methods are tested. The multi-displacement amplification (MDA) method (the REPLI-g kit) that produces relatively long wgaDNA fragments > 10 kb [19], and the OmniPlex method (the GPlex2 and the GPlex4 kits) that produces fragments approximately 500 bp long [20]. We also test the effect of using either one or three 3.2 mm disks and of extracting proteins from the disks before the DNA-extraction. Finally, the robustness of the selected approaches was evaluated.

## Methods

### Subjects

The investigation comprised 24 subjects born between 1982 and 1992, who all had their residual neonatal DBSS stored at -24°C in the DNSB. Four subjects were informed volunteers and 20 were from a genetic study on schizophrenia (ethical approval number: 20020020; data protection agency number: 2002-41-2059).

### DNA-extraction and WGA methods

Reference gDNA was purified from venous blood samples from the four volunteers and from four subjects from the schizophrenia study using the Maxwell 16 automatic system and the Maxwell^® ^16 Blood DNA Purification Kit (Promega). Neonatal DBSS from the eight participants were retrieved from the DNSB, and DNA was extracted from one or three DBSS disks, 3.2 mm in diameter, using Extract-N-Amp Blood PCR Kit (ENA)(extraction volume: 200 μL) (Sigma-Aldrich) or QIAamp DNA Blood Micro Kit (QIA)(extraction volume: 75 μL) (Qiagen). The DNA extracts were amplified using the REPLI-g kit (Qiagen), GenomePlex^® ^Complete WGA Kit (GPlex2, Sigma-Aldrich), or GenomePlex^® ^Single Cell Whole Genome Amplification Kit (GPlex4, Sigma-Aldrich). All procedures were performed according to the manufacturer's instructions. Prior to DNA-extraction, a subset of disks was extracted for proteins as described by Skogstrand et al. 2005 [21]. Please consult Additional file 1 for set up. Furthermore, two DBSS disks from 16 other subjects were extracted for proteins before DNA-extraction using the ENA kit, and the DNA extracts were amplified using the REPLI-g and the GPlex4 kits. DNA was quantified using Quant-iT™ PicoGreen^® ^dsDNA Reagent (Molecular Probes, Invitrogen).

### Genotyping

The gDNA and wgaDNA samples were marked on the Illumina Infinium HD Human610-Quad BeadChip (Illumina) according to the manufacturer's instructions, with the exception that 240 ng of wgaDNA starting material was used instead of the prescribed 200 ng. The BeadChips were scanned using the BeadStation 500GX (Illumina) with a high-density upgrade and an AutoLoader (Illumina). The BeadStudio v.3 software (Illumina Corp.) was used for calculating call- and conflict-rates. In the first part of the study all calls were made using the reference Human610-Quadv1B cluster file from Illumina that is constructed from gDNA. In the second part of the study two cluster files, each constructed from 16 wgaDNA preparations made by the REPLI-g and GPlex4 kits (tailored cluster files specific for WGA method), were also used to analyse the respective samples. Conflict-rates were estimated comparing the wgaDNA samples to their respective reference gDNA samples. The percentage of conflicts introduced due to an allelic dropout (eg. AB to AA) was estimated by re-coding the Illumina data to variables allowing comparison using STATA v.9.0.

## Results

The genotyping performance of the different wgaDNA preparations is seen in Additional file 1. The ENA DNA-extraction combined with the REPLI-g kit WGA featured the highest call-rates (99.30–99.51%) and the lowest conflict-rates (0.02–0.03%).

Combining REPLI-g WGA with QIA DNA-extraction was less successful and the results were highly variable. The genotyping performance using wgaDNA made by the two OmniPlex method kits, GPlex2 and GPlex4, was independent of the DNA-extraction method, with GPlex4 showing consistently higher call rates than GPlex2 (Wilcoxon's paired test, p < 0.001). The reference gDNA call-rates were 99.8–99.9%. Almost all conflicts between results from the wgaDNA preparations and the reference gDNA were due to an allelic dropout (data not shown). Notably, extraction and amplification procedures that produced high call-rates displayed low conflict-rates with the reference gDNA and vice versa, which indicates that genome-wide scans on wgaDNA are reliable when the call-rates are high [Additional file 1]. This was partially confirmed by calculating the correlation coefficients between the call- and conflict-rates of the three WGA kits [Additional file 1]. It made no significant difference whether one or three DBSS disks were used for extraction. No systematic differences in genotyping performance were related to sample age.

**Figure 1:**
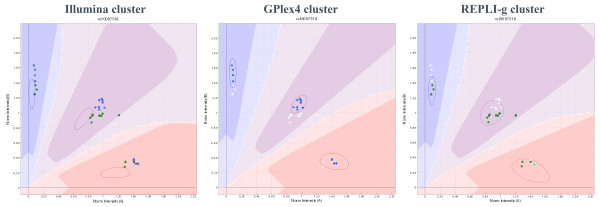
**Plot of the normalized values measure for the A allele and B allele**. The same 16 samples were amplified using the GPlex4 and the REPLI-g WGA kits. The "Illumina cluster" plot shows how the GPlex4 (blue dots) and the REPLI-g (green dots) wgaDNA genotypes compare to the Illumina cluster file. The "GPlex4 cluster" plot shows how a custom-made cluster file based on GPlex4 samples (blue dots) improves both fit and call-rate of the loci. The "REPLI-g cluster" plot shows how a custom-made cluster file based on REPLI-g samples (green dots) improves both fit and call-rate of the loci.

Based on the results displayed in Additional file 1, the combinations of DNA extraction by the ENA kit and WGA by the REPLI-g and GPlex4 kits were selected for further evaluation. For this, 16 new subjects were employed. After DNA-extraction, WGA and subsequent genome-wide scans (GWS), the results were analysed using both a standard Human610-Quadv1B Cluster, provided by Illumina, and WGA kit specific tailored cluster files. The rationale for the tailored cluster files is demonstrated in Figure 1. Generally, the wgaDNA samples cluster nicely, but not always in the area defined by the Illumina Human610-Quadv1B cluster file, which is based on gDNA samples. By creating tailored WGA-specific cluster files and using these for analysis, the genotype call-rates of both set-ups (REPLI-g and GPlex4) improved significantly (Wilcoxon paired test, p < 0.001) as seen in Table 1. Comparison of the call-rates indicated that the REPLI-g samples had a significantly higher call-rate than the GPlex4 samples (Wilcoxon's paired test, p < 0.001). Comparison of the amount of wgaDNA amplified by each kit revealed no significant difference (Wilcoxon's paired test, p > 0.050).

**Table 1:** Robustness of the GPlex4 and REPLI-g WGA kits.

**ID**	**GPlex4**			**REPLI-g**		
	
	**Call-rate A**^1^	**Call-rate B**^2^	**WGAout**^3^	**Call-rate A**^1^	**Call-rate B**^2^	**WGAout**^3^
1	97.74%	99.24%	4.86	99.42%	99.65%	6.55
2	97.41%	99.30%	4.85	99.56%	99.64%	7.22
3	98.21%	99.42%	5.06	98.54%	99.33%	3.53
4	97.80%	99.38%	4.93	99.14%	99.56%	3.00
5	97.90%	99.41%	5.20	99.00%	99.51%	4.47
6	98.04%	99.42%	4.92	99.73%	99.73%	3.74
7	97.83%	99.38%	5.00	99.72%	99.73%	6.02
8	97.62%	99.29%	5.01	99.49%	99.66%	6.00
9	97.56%	99.29%	5.15	99.55%	99.64%	7.64
10	97.30%	99.26%	5.33	95.91%	99.15%	7.86
11	98.17%	99.32%	5.41	99.43%	99.60%	7.48
12	98.13%	99.38%	5.49	99.38%	99.56%	1.56
13	96.62%	99.08%	5.15	99.43%	99.60%	6.71
14	97.55%	99.35%	5.48	99.22%	99.63%	1.40
15	96.75%	99.17%	5.14	98.97%	99.53%	4.98
16	96.53%	*99.16%*	4.13	98.49%	99.43%	2.72

*Median*	*97.68%*	*99.31%*	*5.10*	*99.40%*	*99.60%*	*5.49*
*Std. Dev.*	*0.53%*	*0.10%*	*0.33*	*0.92%*	*0.15%*	*2.18*

^1^Call-rate (percent) using the Illumina Human610-Quadv1B cluster file.

^2^Call-rate (percent) using the WGA kit custom cluster file.

^3^wgaDNA (μg) produced per reaction.

## Discussion

We demonstrate that wgaDNA, made from 3.2 mm disks of DBSS that have been stored at -24°C for more than 20 years is well suited for reliable genotyping of 610,000 SNPs, with call-rates comparable to those obtained using gDNA. The accuracy of genotype calls using wgaDNA from stored DBSS has been of some concern. The issue has been addressed several times, using both low and medium throughput genotyping platforms, and overall with good success [11,12,14-18]. In this study we took the usage of DBSS one step further by conducting GWS. Moreover the accuracy of genotype calls from wgaDNA was assessed by comparing the results with results from high-quality reference gDNA from the same individuals. Initially, we tested two commercial DNA-extraction procedures, three WGA procedures, the effect of number of 3.2 mm disks used, and the effect of protein extraction prior to the gDNA extraction. The efficiency and reliability of the GWS were highly dependent on the employed DNA-extraction and WGA method. Interestingly, call- and conflict-rates were inversely related; indicating that genome scan of wgaDNA is highly reliable when the call-rates are close to 100%. However because only few samples were available to calculate the correlation coefficient, we cannot clearly define a cutoff for the call-rate that would ensure reliable genotyping. In general, the OmniPlex method performed more constantly than the MDA method, producing fairly the same call-and conflict-rates independently of the other variables tested. Of the two OmniPlex based kits the GPlex4 kit performed the best, showing high call-rates and low error-rates. The MDA method performed excellent using the ENA extraction kit and poorly when using the QIA extraction kit. In general, it appeared unimportant whether one or three DBSS disks were used for extraction. This was surprising since the amount of input gDNA for the WGA reactions is supposed to be critical, and in our set-up it was often below the lower limit of 10 ng that is recommended by the manufacturer. Moreover, the preceding protein extraction ofthe disks did not impair the genotyping of the produced wgaDNA, which is in accordance with similar observations from our laboratory [17].

Because the investigation focuses ondifferent combinations of wgaDNA preparation, it suffers from the weakness that the number of samples in each group is limited. In addition, only samples from the DNSB were used.

The combination of the ENA DNA-extraction with either the REPLI-g or the Gplex4 WGA kit were selected to see if the procedures were robust enough for GWA studies employing numerous samples. Both set-ups produced wgaDNA from 16 DBSS stored for 15 to 25 years that performed well with constant high call-rates. Corresponding reference gDNA samples were not available. Notably, when calling genotypes of wgaDNA preparations with the BeadStudio software, albeit clusters were nice and tight for some loci they did not fit well into the standard cluster positions. This is because the BeadStudio software calls the genotypes of a given locus by comparing the observed values with the expected values, defined by the Human610-Quadv1B Cluster file, which is based on gDNA samples [22,23]. In such cases, data fit and call-rates can be improved by adjusting the cluster positions to match the observed data [23]. Cluster files tailored for the OmniPlex and MDA method were hence created from the samples available, and the call-rates were significantly improved for both wgaDNA preparations. They were in fact comparable to call-rates obtained using high-quality gDNA, indicating that the approach is robust.

Eighteen WGA reactions, each producing ~5 μg of wgaDNA, can be made per ENA DNA extraction. As the Illumina Infinium HD Human610-Quad BeadChip uses 240 ng of wgaDNA, one WGA reaction is enough to run 20 chips. Thus one to three 3.2 mm disks from a DBSS are sufficient to make repeated GWS as well as fine-mapping genotyping, if required. We have briefly tested the performance of the two wgaDNA preparations on the Affymetrix platform and found that wgaDNA produced by the OmniPlex method was unsuitable, whereas wgaDNA produced by the MDA method gave results comparable to those obtained by the Illumina platform. In addition to being used for GWS, DBSS can also be used for multiplex protein measurements [21], quantitative RNA micro arrays detecting up to 3000 genes [24], and quantitative DNA methylation analysis [25].

## Conclusion

The results demonstrate that residual DBSS from neonatal screening that have been stored for several years in biobanks can be used for GWS and hence for large genome-wide association studies. Using DBSS instead of collecting new samples may, in a cost-efficient way, reveal important correlations between genotypes, environment and human diseases. Both the OmniPlex and the MDA method performed excellently in combination with the ENA extraction, and we recommend to test which of the two WGA procedures is most suitable for a given task.

## Authors' contributions

MVH prepared the DNA samples, participated in the design of the study and did the major part of data analysis and drafting the manuscript. JG conducted the Illumina GWS and participated in the data analysis. AB, MN, BNP, TFO, PMB, CW, OM, MD and PT initiated the study and participated in its design and the interpretation of results. DMH initiated the study, participated in its design, the interpretation of results and drafting the manuscript. All authors were involved in a critical revising and approved the final manuscript.

## Supplementary Material

Additional file 1 **Performance of different wgaDNA preparations**. 1 Year the DBSS was stored. 2 Kit used for DNA-extraction, QIA-QIAamp DNA Blood Micro Kit; ENA-Extract-N-Amp Blood PCR Kit. 3 Extraction of proteins from DBSS prior to extraction of DNA. 4 Number of 3.2 mm disks used. 5 DNA (ng) utilized per WGA reaction. 6 GWS genotypes call-rate (percent). 7 Rate of conflicts (percent) between genotype results on wgaDNA and reference gDNA. 8 wgaDNA (μg) produced per reaction. Click here for file
