# Supplementary material for: Genome-wide scans using archived neonatal dried blood spot samples
Source: BMC Genomics. 2009 Jul 4;10:297. doi: 10.1186/1471-2164-10-297 (PMC2713266; doi:10.1186/1471-2164-10-297)
Supplement: Additional file 1 — Performance of different wgaDNA preparations. 1 Year the DBSS was stored. 2 Kit used for DNA-extraction, QIA-QIAamp DNA Blood Micro Kit; ENA-Extract-N-Amp Blood PCR Kit. 3 Extraction of proteins from DBSS prior to extraction of DNA. 4 Number of 3.2 mm disks used. 5 DNA (ng) utilized per WGA reaction. 6 GWS genotypes call-rate (percent). 7 Rate of conflicts (percent) between genotype results on wgaDNA and reference gDNA. 8 wgaDNA (μg) produced per reaction. [file 1471-2164-10-297-S1.pdf]

ID - yr.[1]	DNA kit[2]	Ext.[3]	Disk#[4]	DNAin[5]	GPlex 2 Call-rate[6]	GPlex 2 Conf.-rate[7]	GPlex 2 WGAout[8]	GPlex 4 Call-rate[6]	GPlex 4 Conf.-rate[7]	GPlex 4 WGAout[8]	REPLI-g Call-rate[6]	REPLI-g Conf.-rate[7]	REPLI-g WGAout[8]
1 - 1982	QIA	Yes	1	3.8	93.07	0.072	7.41	98.43	0.025	5.39	90.53	0.947	6.10
			3	9.6	96.30	0.027	6.87	98.27	0.025	4.54	98.00	0.073	7.15
2 - 1982	QIA	Yes	1	2.3	94.74	0.080	6.04	98.15	0.029	4.19	84.95	4.365	4.97
			3	10.6	94.72	0.090	6.33	98.33	0.025	5.12	62.91	8.517	4.47
3 - 1992	QIA	Yes	1	7.7	96.73	0.066	6.68	98.09	0.038	4.79	98.41	0.042	5.39
			3	17.3	95.82	0.033	6.11	98.53	0.025	4.14	99.26	0.018	6.64
4 - 1985	QIA	No	1	5.2	96.82	0.044	6.70	97.93	0.033	4.26	19.79	7.626	7.61
			3	15.3	96.52	0.029	6.10	98.47	0.027	4.91	67.45	14.456	7.06
5 - 1989	QIA	No	1	10.3	82.36	0.537	6.30	98.17	0.023	4.74	95.48	0.139	5.46
			3	25.9	97.16	0.023	7.43	98.54	0.021	5.27	84.15	8.696	7.61
6 - 1983	QIA	No	1	5.3	95.47	0.037	5.97	98.20	0.026	4.55	91.58	0.860	4.86
			3	17.6	94.76	0.059	7.27	98.18	0.026	5.18	98.52	0.078	6.47
7 - 1983	ENA	Yes	1	2.6	94.73	0.035	5.71	97.39	0.028	4.90	99.46	0.020	5.43
		No		1.9	94.73	0.039	5.85	96.51	0.048	4.34	99.30	0.030	2.77
8 - 1984	ENA	Yes	1	2.7	96.39	0.040	7.23	97.34	0.049	4.65	99.51	0.031	5.82
		No		3.9	94.39	0.042	6.15	96.78	0.044	3.52	99.30	0.028	2.57
			Median	*6.5*	*95.12*	*0.041*	*6.31*	*98.18*	*0.027*	*4.70*	*96.74*	*0.109*	*5.64*
			Std. Dev.	*7.0*	*3.47*	*0.124*	*0.58*	*0.62*	*0.009*	*0.49*	*21.21*	*4.503*	*1.51*
			Correlation coefficient		*-0.9613*			*-0.8464*			*-0.7066*		

Table 1: Performance of different wgaDNA preparations. [1]Year the DBSS was stored. [2]Kit used for DNA-extraction, QIA- QIAamp DNA Blood Micro Kit; ENA- Extract-N-Amp Blood PCR Kit. [3]Extraction of proteins from DBSS prior to extraction of DNA. [4]Number of 3.2mm disks used. [5]DNA (ng) utilized per WGA reaction. [6]GWS genotypes call-rate (percent). [7]Rate of conflicts (percent) between genotype results on wgaDNA and reference gDNA. [8]wgaDNA (μg) produced per reaction.
